# Implementation status and challenges affecting antimicrobial stewardship programmes in private hospitals in Kampala, Uganda: Insights from a cross-sectional descriptive survey

**DOI:** 10.1371/journal.pgph.0004333

**Published:** 2025-09-25

**Authors:** Doris Kubai, Richard Adome, Edson Munanura, Diane Ashiru-Oredope, Gervason Moriasi

**Affiliations:** 1 Department of Health, County Government of Embu, Embu, Kenya; 2 Department of Pharmacy, School of Health Sciences, Makerere University, Kampala, Uganda; 3 HCAI, Fungal, AMR, AMU and Sepsis Division, United Kingdom Health Security Agency, London, United Kingdom; 4 Department of Biochemistry, ‌‌Microbiology, and Biotechnology, ‌Kenyatta University, Nairobi, Kenya; Federal University Birnin Kebbi, NIGERIA

## Abstract

Private hospitals are critical to healthcare delivery in Kampala, Uganda, where antimicrobial stewardship (AMS) programmes have been introduced as part of the Global Action Plan (GAP) to mitigate the growing threat of antimicrobial resistance (AMR). However, there is limited empirical data on the extent of AMS implementation and the specific barriers these facilities face in adopting such programmes. We therefore evaluated the implementation status and identified key barriers to AMS uptake in private hospitals across Kampala, Uganda. A cross-sectional survey was conducted from 9th January 2024–25th July 2024 in 17 private hospitals, using a mixed-methods approach. Quantitative data were collected using the Commonwealth Partnerships for Antimicrobial Stewardship (CwPAMS) Checklist and analysed descriptively. Qualitative data were gathered through key informant interviews and evaluated using content analysis. The findings revealed significant gaps in AMS implementation. Of the 17 hospitals surveyed, 70.6% (12/17) had not prioritised AMS initiatives in their operations. Besides, 88.2% (15/17) had no budget allocated for AMS activities and 82.4% (14/17) reported insufficient staffing. Additionally, 76.5% (13/17) had not provided AMS-specific training. Furthermore, 64.7% (11/17) lacked regular AMS reports, meeting minutes, or resources for antimicrobial prescribing optimisation, while 41.2% (7/17) had no designated AMS team lead. Besides, qualitative analysis showed structural and institutional challenges, including weak leadership commitment and a lack of sustainable financial planning for AMS programmes. The implementation of AMS programmes in selected private hospitals in Kampala was scanty and constrained by structural, financial, and institutional barriers, including inadequate budget allocation, insufficient staffing, limited training, and the lack of prioritisation of AMS activities. Strengthening institutional support through leadership engagement, funding, and embedding AMS as a core component of hospital policy is essential. Additionally, a multi-stakeholder approach is crucial to driving sustainable AMS adoption, aligning with global AMR mitigation efforts, and ensuring optimal patient outcomes.

## Introduction

Microbial infections remain a significant public health burden, contributing to high morbidity and mortality worldwide, particularly in low- and middle-income countries [1]. Treating these infections is becoming increasingly challenging due to limited access to medicines, diagnostic delays, and the emergence of resistant strains [2]. Antimicrobial resistance (AMR) further complicates these challenges by rendering once-effective drugs useless, leaving healthcare providers with fewer treatment options [3,4]. AMR is primarily driven by the overuse, misuse, and poor regulation of antibiotics in both human and veterinary medicine [5,6]. Without coordinated efforts, AMR will undermine global health gains, leading to longer hospital stays, higher medical costs, and increased mortality [7,8].

Antimicrobial stewardship (AMS) offers a comprehensive approach to address AMR by promoting the responsible use of antimicrobial agents [9,10]. It encompasses clinical guidelines, optimised prescription practices, and educational programmes to support healthcare professionals in balancing effective treatment with resistance prevention [11]. AMS aims to reduce unnecessary antibiotic use, minimise adverse effects, and ensure that antimicrobials remain effective in the long term. Beyond hospitals, AMS also plays a role in outpatient care, nursing homes, and veterinary settings, expanding its impact across healthcare systems [12]. Successful AMS programmes require institutional support, multidisciplinary teams, and continuous monitoring to adapt to evolving resistance patterns [12,13].

The World Health Organisation (WHO) introduced the Global Action Plan (GAP) to help countries develop National Action Plans (NAPs) tailored to their healthcare contexts [14–16]. NAPs provide countries with frameworks to align their strategies with global efforts to combat AMR, fostering collaboration across sectors [12]. Over 100 countries have established NAPs, though progress varies due to resource limitations, infrastructural gaps, and policy challenges [17–21]. Integrating AMS into broader health policies, including universal health coverage, can enhance implementation and sustainability [12]. However, the effectiveness of NAPs hinges on national commitment, funding, and the capacity to translate policies into action [22].

In East Africa, especially Tanzania [23], Kenya [24], and Uganda [25,26], AMS research has mainly focused on public hospitals, with little attention given to private healthcare facilities. This is significant because private hospitals provide critical services, especially in urban areas like Kampala, Uganda, where AMS programmes have recently been introduced. Private hospitals in Uganda, particularly in Kampala, often face insufficient funding for antimicrobial stewardship (AMS) activities, though this does not necessarily reflect an overall lack of resources across all hospital functions. Funding limitations are especially pronounced in the not-for-profit private sector, which relies heavily on donor support, faith-based organisations, and patient fees. For-profit facilities, while generally better resourced, may deprioritise AMS due to perceived limited return on investment [21]. In contrast, the public sector benefits from more direct government involvement, including national support for AMS through Uganda’s National Action Plan on Antimicrobial Resistance (2024/25–2028/29) [27]. However, AMS implementation remains uneven due to broader systemic challenges such as workforce shortages, medicine stock-outs, and limited laboratory capacity. Besides, Uganda has national standards for infection prevention and control (IPC), overseen by the Ministry of Health, with dedicated IPC focal persons at regional referral hospitals [27]. Nonetheless, adherence to these standards varies significantly across both public and private sectors due to gaps in enforcement, resource disparities, and limited institutional capacity [21]. Furthermore, the lack of data on AMS implementation in private hospitals limits the ability to address barriers and improve outcomes. Therefore, this study evaluated the current status of AMS programmes in Kampala’s private hospitals and identified the key obstacles to their success, to guide future interventions.

## Methodology

### Study area

This study was conducted between 9th January 2024 and 25th July 2024 across 17 private hospitals in Kampala, Uganda. These included 12 for-profit and five not-for-profit hospitals. All the hospitals were selected from the 19 private hospitals listed in the Uganda Ministry of Health Uganda Facility Master List, based on their provision of formal written consent to participate in the study. Of the 17 participating hospitals, seven were classified as tertiary hospitals, providing specialised and sub-specialised services with advanced diagnostic and treatment capabilities. Six were secondary-level hospitals offering general inpatient and outpatient care, surgical services, and limited specialist services. The remaining four hospitals were private referral hospitals that received patients from lower-tier health centres for further evaluation and management. These hosipitals had inpatient capacities ranging from 50 to 250 beds, and served diverse urban and peri-urban populations. The not-for-profit hospitals were typically larger, faith-based hospitals with long-standing reputations for affordable and comprehensive care, while the for-profit hospitals catered to a mixed patient population, including middle- and upper-income individuals, often providing more specialised or premium services. No primary-level or community clinics were included in the study.

### Study design

This study employed a cross-sectional survey using a mixed-methods approach to generate both quantitative and qualitative data on AMS implementation in private hospitals in Kampala, Uganda [28,29]. Quantitative data were collected using the Commonwealth Partnerships for Antimicrobial Stewardship (CwPAMS) AMS Checklist Tool. This tool, developed through a modified Delphi consensus process involving seven CwPAMS partnerships and six national contacts in Ghana and Uganda, was designed to assess AMS strategies in healthcare institutions in low- and middle-income countries, based on internationally validated indicators [30]. Although previously applied in 12 public hospitals in Uganda, this study marks its first application in private hospital settings. One checklist was completed per hospital and typically administered during engagements with the hospital’s Medicines and Therapeutics ‌‌Committee.

Qualitative data was collected through key informant interviews to explore institutional-level AMS practices, governance structures, available resources, and barriers to implementation. Participants were purposefully selected to capture a range of expert perspectives and included hospital pharmacists, prescribing physicians, and Medicines and Therapeutics Committee members, as well as two national-level informants: the Head of Quality Assurance at the Ministry of Health and the Head of Pharmacovigilance at the National Drug Authority. A total of 19 in-depth interviews were conducted, one from each private hospital (n = 17), and two from national regulatory institutions, by the lead investigator and trained research assistants with prior qualitative research experience. Interviewees were not necessarily the same individuals who contributed to the quantitative checklist, but all were experienced and involved in AMS activities or hospital governance. Interview participants were recruited via formal invitations through hospital administrators and national institutional contacts. A semi-structured interview guide with open-ended questions was used to elicit in-depth responses tailored to the Ugandan healthcare context. All interviews were conducted in English, audio-recorded with consent, and transcribed verbatim for coding and thematic analysis. To ensure trustworthiness of the qualitative data, credibility was supported through triangulation of multiple data sources and iterative probing during interviews. Transferability was addressed by providing detailed descriptions of institutional settings and participant roles. Dependability was ensured by using a standardised interview guide and audit trails documenting the data collection process. Confirmability was maintained by keeping reflexive field notes and conducting peer debriefing sessions among the research team to minimise interpretive bias.

### Quality control of the data

To ensure reliability, the CwPAMS checklist was pretested in two private hospitals outside Kampala, and the obtained results were considered in refining the data collection tool before deployment in the main study. During data collection, trained research assistants reviewed all completed checklists to confirm that every section was accurately filled. The audio-recorded key informant interviews were transcribed verbatim and cross-verified by independent proofreaders for accuracy, to ensure consistency between recorded content and transcripts, and minimise transcription errors. Any ambiguities were resolved through consultation with the research team to maintain data integrity.

### Data handling and analysis

Quantitative data from the CwPAMS checklist was analysed using descriptive statistics, and the results were expressed as frequencies and percentages to summarise the status of AMS implementation across hospitals. The qualitative data from key informant interviews was analysed using thematic analysis, which is a flexible method for identifying, analysing, and reporting patterns (themes) within the data. The process involved familiarisation with the transcripts, generation of initial codes, searching for and reviewing themes, and defining and naming them. This approach allowed the us to systematically capture contextual nuances, recurring perceptions, and shared experiences regarding AMS practices. A comparative analysis of the statements was also conducted to reveal common challenges and gaps across the hospitals, thus deepening the understanding of the barriers hindering successful AMS implementation.

### Ethical considerations

Ethical approval for this study was obtained from the Makerere University School of Health Sciences Research Ethics Committee (MAKSHSREC-2020–23) and the Uganda National Council of Science and Technology. Informed consent was obtained from all participants following thorough explanations of the study’s purpose and procedures. Hospital administrators were engaged before the study began to ensure alignment with institutional policies. All data was handled with strict confidentiality to protect participants’ privacy. Additional information regarding the ethical, cultural, and scientific considerations specific to inclusivity in global research is included in the Supporting Information (S1 Checklist).

## Results

### Characteristics of the studied private hospitals

Out of the 19 private hospitals invited to participate, 17 consented, yielding an overall response rate of 89.5% (17/19). The participating hospitals comprised both academic and non-academic institutions, with 67.3% identified as teaching hospitals and the remaining 32.7% as non-teaching facilities.

### Administrative and financial support for AMS programmes

The study revealed that 70.6% of the hospitals had not formally integrated AMS into their institutional strategies or included it in their key performance indicators (KPIs) (Table 1). Budgetary limitations were also a significant barrier, as 88.2% of hospitals lacked dedicated, sustainable funding to support AMS activities. Additionally, these hospitals had no documentation to guide AMS operations by defining roles, responsibilities, and collaborative frameworks within AMS teams. Staffing limitations were pronounced, with 82.4% of hospitals reporting insufficient personnel to support AMS efforts effectively (Table 1).

**Table 1:** Senior management leadership towards AMS programmes in private hospitals in Kampala, Uganda, between 9th January 2024 and 25th July 2024 (N = 17).

Statement	Yes n (%)	No n (%)	Somewhat n (%)
Has your hospital management formally identified AMS as a priority objective for the institution and included it in its key performance indicators?	5 (29.4)	12 (70.6)	0 (0.0)
Is there dedicated, sustainable and sufficient budgeted financial support for AMS activities (e.g., support for salary, training, or IT (information technology) support)?	2 (11.8)	15 (88.2)	0 (0.0)
Does your hospital follow any (national or international) staffing standards for AMS activities (e.g., number of full-time equivalent (FTE) per 100 beds for the different members of the AMS team)? (confirm for the standard staffing documents)	2 (11.8)	14 (82.4)	1 (5.9)

### Committee structures and multidisciplinary collaboration

AMS implementation varied across the hospitals, with 64.7% reporting that AMS committees did not consistently document the minutes of their meetings, limiting transparency and progress tracking (Table 2). Despite these challenges, 58.8% of the hospitals had established multidisciplinary AMS teams led by designated leaders, with involvement from clinicians, nurses, or pharmacists beyond those serving directly on AMS committees (Table 2). Medicines and Therapeutics Committees were active in 47.1% of hospitals, although fewer hospitals had IPC and patient safety committees (17.6%) (Table 2). Two hospitals (11.8%) had additional pharmacy-focused committees to oversee AMS initiatives, while only one hospital (5.9%) had a pharmacovigilance committee dedicated to the appropriate use of antimicrobial agents (Table 2). Moreover, 76.5% had no formal documentation outlining areas of collaboration between AMS teams and infection prevention and control (IPC) teams (Table 2).

**Table 2:** Accountability and responsibility for AMS and IPC activities in private hospitals in Kampala, Uganda, between 9th January 2024 and 25th July 2024 (N = 17).

Statement	Yes n (%)	No n (%)	Somewhat n (%)
Does your hospital have a formal/written AMS programme/strategy accountable for ensuring appropriate antimicrobial use?	5 (29.4)	12 (70.5)	0 (0.0)
Does your hospital have a formal organizational multidisciplinary structure responsible for AMS (e.g., a committee that focuses on OR takes responsibility for appropriate antimicrobial use, pharmacy committee, patient safety committee, infection prevention and control committee or other relevant structure)?	8 (47.1)	8 (47.1)	1 (5.9)
Is there a healthcare professional identified as a leader for AMS activities at your hospital and responsible for implementing the programme?	10 (58.8)	7 (41.2)	0 (0.0)
Is a multidisciplinary AMS team available at your hospital (e.g., greater than one trained staff member supporting clinical decisions to ensure appropriate antimicrobial use) to implement your stewardship strategy?	10 (58.8)	7 (41.2)	0 (0.0)
Is there a document clearly defining roles, procedures of collaboration and responsibilities of the AMS team members?	2 (11.7)	15 (88.3)	0 (0.0)
Are clinicians, nurses, or pharmacists, other than those part of the AMS team (e.g., from the ICU, Internal Medicine and Surgery) involved in the AMS committee?	10 (58.8)	6 (35.3)	1 (5.9)
Does the AMS committee produce a regular (e.g., every 6 months) dedicated report which includes, e.g., antimicrobial use data and/or prescription improvement initiatives, with time-committed short term and long-term measurable goals/targets for optimizing antimicrobial use?	2 (11.8)	11 (64.7)	4 (23.5)
Is there a document clearly defining the procedures of collaboration of the AMS team/committee with the infection prevention and control team/committee?	3 (17.6)	13 (76.5)	1 (5.9)
Are minutes or notes taken during the meetings?	5 (29.4)	11 (64.7)	1 (5.9)

### Composition of AMS teams, meeting frequency, training opportunities and infection management expertise availability

#### AMS team composition, leadership, and responsibilities.

Across the 17 surveyed private hospitals, AMS teams included clinicians in 11 hospitals (64.7%), pharmacists in 10 (58.8%), and medical laboratory scientists in 9 (52.9%), indicating strong representation of key clinical and diagnostic personnel. Nurses were included in 8 hospitals (47.1%), infection prevention and control (IPC) officers in 7 (41.2%), and surgeons in 6 (35.3%), reflecting a moderately multidisciplinary approach to AMS. However, only 3 hospitals (17.6%) reported including data analysts as part of the AMS team, revealing a significant gap in technical capacity for data monitoring and interpretation.

In terms of leadership, pharmacists served as AMS leads in 8 hospitals (47.1%), senior clinicians or medical directors in 5 (29.4%), and IPC officers in 3 (17.6%). In one hospital (5.9%), the AMS programme was coordinated by a hospital administrator due to the absence of designated clinical leadership. The key responsibilities of AMS leads typically included organising stewardship meetings, coordinating antimicrobial prescribing audits, supporting IPC activities, and reporting antibiotic consumption and resistance trends to hospital management.

#### AMS meeting frequency.

The frequency of AMS team meetings varied considerably. Six hospitals (35.3%) held meetings on a quarterly basis, four (23.5%) met monthly, and one (5.9%) convened weekly. The remaining six hospitals (35.3%) reported irregular meetings or lacked documented meeting schedules. This inconsistency may contribute to reduced accountability and diminished continuity in AMS programme implementation.

#### AMS training opportunities, infection management expertise, and gaps.

Across the 17 private hospitals included in the study, the frequency, modality, coverage, and sources of AMS training varied considerably. Regarding training frequency, six hospitals (35.3%) conducted AMS training up to four times per year, two hospitals (11.8%) held training sessions biannually, and three hospitals (17.6%) offered training more than four times annually. Four hospitals (23.5%) reported that no AMS or infection prevention and control (IPC) training had been conducted for clinical staff in the past year. In addition, two hospitals (11.8%) had no formal documentation on the frequency or occurrence of AMS training, pointing to weak tracking systems.

Online platforms were the predominant mode of AMS training delivery, used in 13 hospitals (76.5%), while four hospitals (23.5%) conducted face-to-face, in-person sessions. In seven hospitals (41.2%), Continuous Medical Education (CME) sessions were conducted internally by facility staff, indicating a facility-based approach to AMS capacity building. Besides six hospitals (35.3%) reported that pharmaceutical companies facilitated AMS training sessions. Two hospitals (11.8%) received training support from national institutions, including the Ministry of Health, the National Drug Authority (NDA), and the Pharmaceutical Society of Uganda (PSU).

In terms of infection management capacity, all 17 hospitals (100%) reported having access to laboratory and imaging services to support the diagnosis of common infections. All also confirmed that diagnostic results were available in a timely manner to support clinical decision-making. Furthermore, 16 hospitals (94.1%) reported having access to healthcare professionals (e.g., medical doctors, pharmacists, nurses) with training or experience in infection diagnosis, prevention, treatment, and stewardship who were willing to actively participate in AMS activities. One hospital (5.9%) acknowledged the presence of relevant professionals but reported lack of willingness to engage in AMS initiatives, suggesting possible institutional or motivational barriers to effective stewardship implementation.

With regard to IPC training, more than ten doctors and surgeons had been trained in six hospitals (35.3%), and over ten nurses had received IPC training in seven hospitals (41.2%). Pharmacists had undergone IPC training in ten hospitals (58.9%). However, in six hospitals (35.3%), no other clinical staff, such as laboratory personnel, allied health workers, or support personnel, had received any IPC training during the past year.

Despite the presence of various training initiatives, significant training gaps across clinical cadres were identified. In seven hospitals (41.2%), doctors, surgeons, and pharmacists had not received any AMS training in the past year. Nursing staff were similarly undertrained, with eight hospitals (47.1%) reporting that no nurses had received AMS training during the same period. Additionally, nine hospitals (52.9%) indicated that other clinical personnel, including allied health professionals, laboratory scientists, and support staff, had not undergone AMS training. Only four hospitals (23.5%) reported that more than ten doctors and surgeons had received AMS training within the last year. In contrast, pharmacists had participated in AMS training in nine hospitals (52.9%).

### Monitoring, reporting, and feedback mechanisms for AMS activities

The monitoring and surveillance of AMS activities across the studied private hospitals revealed significant gaps. Nearly half of the hospitals (47.1%) tracked the quantity of antimicrobials prescribed, dispensed, or purchased at the unit or hospital level, indicating some level of oversight (Table 3). However, 76.5% of the hospitals acknowledged that their stewardship programmes did not monitor adherence to one or more of the interventions implemented by the AMS teams, suggesting challenges in ensuring compliance with established protocols (Table 3). Additionally, while three private hospitals (17.7%) reported monitoring the antibiotic susceptibility patterns of key bacterial pathogens, none had conducted a point prevalence survey (PPS) on antimicrobial use in the preceding year (Table 3).

**Table 3:** Monitoring and surveillance of AMS activities in private hospitals in Kampala, Uganda, between 9th January 2024 and 25th July 2024 (N = 17).

Statement	Yes n (%)	No n (%)	Somewhat n (%)
Does your hospital monitor the quantity of antimicrobials prescribed/ dispensed/purchased at the unit and/or hospital wide level?	8 (47.1)	8 (47.1)	1 (5.9)
Does your stewardship programme monitor compliance with one or more of the specific interventions put in place by the stewardship team (e.g., indication captured in the medical record for all antimicrobial prescriptions, or antibiotic prescribed follows hospital guidelines)?	2 (16.7)	13 (76.5)	2 (16.7)
Does your hospital monitor antibiotic susceptibility rates for a range of key bacteria?	3(17.6)	13 (76.5)	1(5.9)
Has your hospital conducted a point prevalence survey (PPS) for antimicrobial use in the last year?	0 (0.0)	17(100.0)	0 (0.0)

The absence of comprehensive reporting structures was also evident, with 76.5% of the hospitals lacking hospital-specific reports on the volume of antimicrobials used (Table 4). Consequently, these hospitals did not provide prescribers with feedback on prescribing patterns, hindering efforts to optimise antimicrobial use. Similarly, 70.6% of the hospitals did not share facility-specific reports on antibiotic susceptibility patterns with healthcare providers, limiting their ability to make data-informed treatment decisions (Table 4). Furthermore, 64.7% of the hospitals did not communicate the findings of audits or reviews on the quality and appropriateness of antimicrobial use to prescribers, impairing efforts to foster continuous improvement (Table 4).

**Table 4:** Reporting and feedback on AMS activities in private hospitals in Kampala, Uganda, between 9th January 2024 and 25th July 2024 (N = 17).

Statement	Yes n (%)	Non (%)	Somewhat n (%)
Are hospital-specific reports on the quantity of antimicrobials prescribed/dispensed/purchased shared with/feedback to prescribers?	4 (23.5)	13 (76.5)	0 (0.0)
Does your stewardship programme share facility specific reports on antibiotic susceptibility rates with prescribers?	4 (23.5)	12 (70.6)	1 (5.9)
Are results of audits/reviews of the quality/ appropriateness of antimicrobial use communicated directly with prescribers?	4 (23.5)	11 (64.7)	2 (11.8)

### Actions to promote responsible antimicrobial use and combat AMR

Efforts to promote responsible antimicrobial use varied across the hospitals, with notable disparities in the implementation of key stewardship strategies. Most of the private hospitals (70.6%) reported having up-to-date recommendations for infection management, covering the diagnosis, prevention, and treatment of infectious diseases (Table 5). However, only 47.1% had established antimicrobial stewardship (AMS) protocols, such as restricted antimicrobial formularies and intravenous (IV)-to-oral switch policies, which had been ratified by their hospital administrations. Infection prevention and control (IPC) protocols, including hand hygiene practices and water, sanitation, and hygiene (WASH) measures, had been formalised in 88.2% of the hospitals, reflecting a strong administrative commitment to IPC efforts (Table 5). Nonetheless, regular IPC ward rounds were absent in 64.7% of the hospitals, potentially limiting real-time surveillance and infection control activities (Table 5). Local antimicrobial prescribing guidelines tailored to hospital-specific needs were in place in 70.6% of the hospitals, reinforcing the importance of context-specific antibiotic use strategies (Table 5). However, these guidelines were absent in 23.5% of the hospitals with one hospital (5.9%) unclear of their availability (Table 5).

**Table 5:** Other actions aimed at responsible antimicrobial use in private hospitals in Kampala, Uganda, between 9th January 2024 and 25th July 2024 (N = 17).

Statement	Yes n (%)	No n (%)	Somewhat n (%)
Does your hospital have available and up-to-date recommendations for infection management (Diagnosis, prevention and treatment)?	12 (70.6)	4 (23.5)	1 (5.9)
Do you have any published AMS protocols, e.g., restricted antimicrobial list, IV to oral policy (that have been ratified for use within your hospital)?	8 (47.1)	9 (52.9)	0 (0.0)
Do you have any published Infection Prevention and Control protocols, e.g., hand hygiene, WASH (that have been ratified for use in your health institution)?	15 (88.2)	2 (11.8)	0 (0.0)
Are there regular infection and antimicrobial prescribing focused ward rounds in specific departments in your hospital?	6 (35.3)	11 (64.7)	0 (0.0)
Does the hospital have local/hospital specific antimicrobial prescribing guidelines? This may be included as part of a wider drug formulary	12 (70.6)	4 (23.5)	1 (5.9)
If yes, do these guidelines include WHO AWaRe* categories?	6 (50.0)	6 (50.0)	0 (0.0)

Besides, while 29.4% of hospitals reported using at least three tools to support AMS activities, another 35.3% had access to only two guidelines, while 5.9% of the hospitals had only one tool. Alarmingly, 29.4% of hospitals reported having no tools or guidelines in place to support AMS or IPC initiatives.

### Hospital initiatives focusing on antibiotic use awareness and AMR to foster AMS

Awareness of national or regional infection management guidelines was reported in 82.4% of the hospitals, indicating relatively strong engagement with higher-level policy frameworks. However, awareness of national campaigns on AMR was limited; 58.8% of the respondents were unaware of any national newspaper campaigns on antibiotic awareness, and 52.9% reported no familiarity with the World Antibiotics Awareness Week (Table 6). Additionally, 94.1% of the hospitals had not encountered any AMR initiatives beyond those listed in the survey checklist, except for one respondent who had received information on AMR from pharmaceutical representatives (Table 6).

**Table 6:** Initiatives within private hospitals in Kampala or in the republic of Uganda which focus on antibiotic awareness and resistance between 9th January 2024 and 25th July 2024 (N = 17).

Initiatives	Respondent Awareness	
	Yes, n(%)	No, n(%)
TV or radio advertising for the public	4 (23.5)	13 (76.5)
Toolkits and resources for healthcare workers	10 (58.8)	7 (41.2)
National or regional guidelines on management of infections	14 (82.4)	3 (17.6)
Awareness raising from professional organisations	11 (64.7)	6 (35.3)
Conferences/events focused on tackling antibiotic resistance	11 (64.7)	6 (35.3)
National or regional posters or leaflets on antibiotic awareness	9 (52.9)	8 (47.1)
Newspaper (national) articles on antibiotic resistance	7 (41.2)	10 (58.8)
National campaign	7 (41.2)	10 (58.8)
World Antibiotic Awareness Week	8 (47.1)	9 (52.9)
Water Sanitation and Health (WASH) initiatives with Global Hand Hygiene/WHO	11 (64.7)	6 (35.3)
I am not aware of any initiatives	1 (5.9)	16 (94.1)
Others (Commissioning of a Drugs and Therapeutics Committee)	1 (5.9)	16 (94.1)

Respondents in 64.7% of hospitals reported receiving awareness about AMR from professional organisations, conferences, and global initiatives such as WHO campaigns on hand hygiene and WASH (Table 6). Television or radio channels served as awareness platforms for only 23.5% of the hospitals, highlighting gaps in public-facing AMR campaigns (Table 6).

In terms of concrete actions, 58.8% of the hospitals reported having implemented key measures in the previous year to promote responsible antibiotic use and combat AMR. These actions included AMS training, establishing pre-authorisation drug lists, conducting peer review workshops on prescribing practices, and offering prescribers mandatory feedback on their prescribing habits. Additional efforts involved restricting access to certain medications, ensuring only authorised clinicians issued prescriptions, requiring pre-treatment laboratory tests, and enhancing laboratory capacity for culture and sensitivity testing. Some hospitals also strengthened the application of antibiotic prescribing guidelines and monitored antimicrobial consumption via pharmacy departments. One hospital (5.9%) reported the recent commissioning of a Drugs and Therapeutics Committee as a critical intervention to oversee medicine use and address AMR (Table 6). In contrast, 35.3% of hospitals had not undertaken any specific actions within the past year to address AMR or promote judicious antimicrobial use. One respondent (5.9%) expressed uncertainty regarding whether any such actions had been taken in their institution (Table 6).

### Barriers to effective AMS implementation in the studied private hospitals

Several barriers were identified as key impediments to the successful implementation of AMS programmes in the studied private healthcare facilities in Kampala, Uganda. A lack of sufficient time for qualified personnel to engage in AMS activities was reported by 76.5% of hospitals (Table 7). Inadequate resource allocation for AMS initiatives was cited by 58.8% of respondents, reflecting structural challenges in prioritising and sustaining AMS programmes within private healthcare settings (Table 7).

**Table 7:** Barriers to the implementation of AMS programs in private hospitals in Kampala, Uganda between 9th January 2024 and 25th July 2024 (N = 17).

Main barriers to AMS in Private Hospitals	n (%)
Lack of qualified personnel	1 (5.9)
Qualified personnel do not have enough time to perform stewardship	13 (76.5)
Lack of motivated or engaged staff	5 (29.4)
Lack of funding	10 (58.8)
Lack of support from hospital management	3 (17.6)
Insufficient microbiology laboratory capacity	5 (29.4)
Inadequate use of the microbiology laboratory	2 (11.8)
Poor quality of antibiotics	7 (41.2)
Regular shortages or stock-outs of essential (“Access”) antibiotics	3 (17.6)
High cost of antibiotics	8 (47.1)
Lack or unavailability of practical, evidence-based, local guidelines	7 (41.2)
Lack of trust in local guidelines	3 (17.6)
Lack of cooperation from prescribers	7 (41.2)
Lack of knowledge on good prescribing practices among clinicians	4 (23.5)
Lack of expertise and training in AMS within the AMS team	6 (35.3)
Lack of confidence in hospital infection prevention and control (IPC) processes	0 (0.0)
Lack of information technology support	4 (23.5)
Patient demands or belief	9 (52.9)
Other	2 (11.8)

Additionally, healthcare workers’ inclination to acquiesce to patient demands or adhere to prevailing patient beliefs was identified as a significant barrier by 52.9% of hospitals (Table 7). Furthermore, the high cost of certain antibiotics, reported by 47.1% of hospitals, was recognised as a critical barrier, particularly in cases where the financial burden limited access to optimal treatment options or discouraged the use of recommended antimicrobial therapies (Table 7).

### Qualitative insights from key informant interviews

The qualitative component of this study yielded five overarching themes that reflect the current state of antimicrobial stewardship (AMS) implementation in private healthcare settings in Kampala: (1) heterogeneous awareness of AMS concepts; (2) weak reporting and governance structures; (3) limited regulatory oversight and monitoring; (4) presence but underutilisation of policy frameworks; and (5) contextual barriers to effective AMS implementation in the private sector.

#### Theme 1: Heterogeneous awareness of AMS concepts.

The interviewees demonstrated variable familiarity with AMS. While some hospital-based clinicians and pharmacists were conversant with the objectives and principles of AMS, others, especially those without direct involvement in stewardship activities, lacked basic awareness.

“AMS is about promoting rational use of antimicrobials to reduce resistance and treatment costs.” — *Hospital pharmacist, private facility.*

Conversely, others acknowledged unfamiliarity with the concept, despite recognising the importance of responsible antimicrobial use:

“*Honestly, I’m hearing about AMS for the first time. But I think it means ensuring we don’t misuse antibiotics and contribute to resistance*.” — *General physician, private hospital.*

#### Theme 2: Weak reporting and governance structures.

The absence of a formalised AMS reporting system within private hospitals emerged as a consistent finding. Most facilities did not submit stewardship-related data to national regulatory bodies, citing lack of policy enforcement and limited institutional capacity.

“*There’s no requirement for private hospitals to report AMS indicators. They mainly submit reports on general medicine use*.” — *Official, Ministry of Health.*

Additionally, non-functional or inactive Medicines and Therapeutics Committees were reported as a significant barrier to AMS governance at the facility level.

“*Medicines and Therapeutics Committees exist on paper, but many are inactive, which makes AMS monitoring difficult*.” — *Hospital administrator, private sector.*

#### Theme 3: Limited monitoring and evaluation by regulatory agencies.

At the national level, AMS-related data are primarily collected reactively, often triggered by pharmacovigilance reports rather than through systematic monitoring.

“*We mostly receive information on antimicrobials when complaints are submitted—say a drug isn’t working. Then we investigate further*.” — *Senior pharmacovigilance officer, National Drug Authority (NDA).*

For instance, a recent investigation into reports of ceftriaxone treatment failure revealed irrational prescribing practices rather than poor drug quality:

“*Ceftriaxone passed all quality tests. The issue was irrational use—wrong dosing and incomplete regimens*.” — *Regulatory scientist, NDA.*

While quality improvement teams have been established in health facilities to support data-driven stewardship, their functionality varies widely, and structured AMS monitoring remains limited in the private sector.

“*Some facilities have quality improvement teams, but they lack clear mandates or the capacity to monitor AMS consistently*.” — *Quality assurance lead, Ministry of Health.*

#### Theme 4: Policy frameworks and guidelines: available but underutilised.

National guidelines supporting AMS are in place and applicable to both public and private facilities. These include the Uganda Clinical Guidelines (UCG), the National Infection Prevention and Control (IPC) Guidelines (2013), and the National Action Plan on Antimicrobial Resistance (AMR-NAP, 2018–2023). However, implementation and adherence remain suboptimal in the private sector.

“UCGs and IPC guidelines are available to all prescribers. But enforcement and uptake in private hospitals are not always consistent.” — *Policy advisor, Ministry of Health.*

The National Drug Authority (NDA) also supports stewardship through its regulatory role in antimicrobial licensing, importation, and facility accreditation.

“NDA oversees regulation of antimicrobials, and we’re involved in initiatives like SPARS to assess facility performance.” — *Senior regulator, NDA.*

#### Theme 5: Contextual challenges in private healthcare facilities.

Several barriers unique to the private sector were identified, including high costs of diagnostics, profit-driven prescribing behaviours, and patient-driven demands for specific antimicrobials.

“*Culture and sensitivity tests are expensive, so hospitals or patients often skip them*.” — *Clinical pharmacist, private hospital.*

“*Private facilities face pressure to generate income, which sometimes leads to prescribing antibiotics not based on need but profitability*.” — *Medical officer, private facility.*

Prescribers also described challenges in managing patient expectations, especially when patients insist on specific antimicrobials based on prior experiences or perceived effectiveness.

“*Patients sometimes demand certain antibiotics. Clinicians give in just to keep them satisfied—even when it’s not indicated*.” — *Prescribing physician, private hospital.*

“*Balancing business interests with appropriate prescribing is a major challenge in private hospitals, unlike the public sector, where we follow national supply guidelines*.” — *Medicines and Therapeutics Committee member, private facility.*

## Discussion

This study assessed the implementation status of antimicrobial stewardship programmes (ASPs) in private hospitals in Kampala, Uganda, highlighting key barriers and opportunities to foster effective implementation. The findings indicate that AMS is often not prioritised by hospital management, with limited budget allocations and resources constraining implementation. Additionally, poor coordination between antimicrobial stewardship (AMS) and infection prevention and control (IPC) teams, alongside high antibiotic costs and patient expectations, were the most critical challenges. The integration of insights from healthcare staff and key informants from the National Drug Authority (NDA) and the Ministry of Health enriches our understanding of the practical difficulties faced. This study also underscores the importance of aligning hospital-level AMS strategies with national AMR action plans to create synergistic effects that foster sustainability.

In this study, most qualified personnel cited a lack of time for AMS activities and inadequate resource allocation, reflecting its low prioritisation within hospital leadership. Similar to findings elsewhere, hospitals with insufficient budgets struggle to sustain AMS programmes, often lacking essential diagnostics and defaulting to empirical use of broad-spectrum antibiotics, which exacerbates inappropriate prescribing and resistance [31–33]. This lack of prioritisation also undermines staff motivation, as insufficient support and limited capacity-building opportunities weaken engagement in AMS efforts [34,35]. Establishing clear AMS operational frameworks with defined objectives, roles, and timelines would promote accountability and structured implementation [34]. Furthermore, linking AMS to performance indicators and incentivising compliance through grants or subsidies from national health authorities could reinforce commitment and integrate stewardship into long-term institutional planning [36]. Together, these measures would strengthen AMS governance, optimise resource allocation, and improve hospital-wide participation.

Despite national guidelines such as the Uganda Clinical Guidelines (UCG) and the AMR National Action Plan (AMR-NAP), the disconnect between policy and hospital-level implementation was evident. The findings reflect the structural challenge where National AMR policies remain abstract and are not translated into actionable activities at the hospital level [35]. Evidence from other low- and middle-income countries demonstrates that aligning AMS with hospital performance metrics improves adherence to protocols and sustains stewardship initiatives [12,36]. A key strategy to address this gap involves creating tailored AMS action plans specific to each hospital, ensuring that policies are contextualised and aligned with operational realities [37]. Integrating AMS into hospital accreditation frameworks may further encourage management to prioritise stewardship activities by linking compliance to financial and reputational incentives [38].

The absence of structured collaboration between AMS and IPC teams emerged as a major barrier in private hospitals, with most respondents reporting non-functional or absent partnerships, thereby limiting the effectiveness of both programmes. Evidence shows that integrated AMS–IPC frameworks improve patient outcomes, reduce antibiotic use, and lower healthcare-associated infections, whereas fragmented approaches result in duplication, missed opportunities, and delayed outbreak detection [36,39–41]. Establishing cross-functional AMS–IPC committees with defined roles, regular data review meetings, and shared action plans would facilitate coordinated interventions such as optimising surgical prophylaxis, improving diagnostic stewardship, and enhancing resistance surveillance [39,42]. Strengthening communication channels and aligning monitoring systems would also enable earlier detection of resistant infections and data-driven decision-making [39,42]. Insights from key informants emphasised the need for collaboration with regulatory authorities to ensure timely guidance on AMR trends and best practices; while leveraging digital tools such as integrated dashboards could further improve real-time data sharing and multidisciplinary engagement [43,44]. Such measures would foster accountability and sustain effective AMS–IPC integration across private hospitals.

This study revealed significant inconsistencies in AMS training, with inadequate frequency and comprehensiveness contributing to knowledge gaps and hindering standardised prescribing practices [37]. Continuous professional development, incorporating targeted workshops and refresher courses tailored to specific cadres, is essential to improve AMS competencies [45]. Patient demand also influenced prescribing in over half of cases, underscoring the need for patient education campaigns to reduce inappropriate antibiotic use and raise awareness of resistance risks [46–48]. Integrating AMR education into hospital engagement strategies and leveraging media platforms could strengthen public participation in stewardship [49,50]. Furthermore, feedback mechanisms, including peer review, mentorship, and digital tools for real-time prescribing audits, would enhance accountability and evidence-based practice [44,47,51]. Broader capacity building, through investments in diagnostics, data systems, and supply chains, alongside partnerships with academic institutions and hospital networks, would further support sustainable AMS implementation [42,45,50,52].

This study identified several barriers to AMS implementation, including insufficient resources, high antibiotic costs, limited diagnostic capacity, and patient expectations, reflecting systemic challenges typical of low- and middle-income countries where competing priorities hinder stewardship efforts [43,53,54]. The lack of diagnostics often drives empirical prescribing, increasing inappropriate antimicrobial use, while investments in rapid diagnostic tools and partnerships with public health laboratories could facilitate evidence-based decision-making and strengthen AMS [11,12,31,41]. The high cost of antibiotics further limits access to targeted therapies, forcing reliance on broad-spectrum agents, with pricing shown to influence prescribing choices and contribute to suboptimal treatment [32,55]. Pooled procurement models, bulk purchasing, and government subsidies could mitigate cost barriers, while collaboration with pharmaceutical companies on cost-sharing may improve access but requires oversight to prevent conflicts of interest and ensure training remains evidence-based and free from commercial bias [56,57].

Patient expectations for antibiotics also emerged as a major barrier, with clinicians frequently prescribing to meet demands, undermining stewardship efforts. Evidence demonstrates that targeted patient education campaigns reduce inappropriate antibiotic use by managing expectations and raising awareness of resistance risks [58,59]. Embedding AMR education into routine consultations and implementing shared decision-making frameworks can empower patients to participate in stewardship-focused care [59,60]. Hospitals should integrate patient-centred communication strategies with broader public awareness campaigns to reinforce appropriate antibiotic use and align treatment decisions with AMS objectives. These combined approaches, addressing systemic, economic, and behavioural barriers, are essential to strengthening stewardship and optimising antimicrobial use in resource-limited settings [5,13,44,61].

Monitoring and evaluation (M&E) systems were identified as major weaknesses, with inadequate surveillance and reporting hindering comprehensive assessment of antimicrobial use [41,62]. The absence of standardised tools in private hospitals limits meaningful comparisons, while integrating digital platforms for automated data collection and centralised antimicrobial use databases could enhance transparency and coordination with national reporting systems [12,21,22]. Regular audits, feedback to prescribers, and benchmarking against peer institutions would strengthen accountability and drive continuous improvement [45,52]. Linking AMS performance indicators to accreditation and funding, alongside establishing multidisciplinary M&E committees equipped with real-time analytics, would further optimise antimicrobial use [35,37,50]. Addressing these gaps requires investment in human resources, infrastructure, and technical expertise, supported by partnerships with research institutions to build capacity in data analysis and evidence-based decision-making [51,63–65]. Such strengthened M&E frameworks would create a dynamic feedback loop, enabling hospitals to refine AMS interventions and respond proactively to evolving resistance patterns

## Study limitations

This study has some limitations that may affect the generalisability of the findings. Firstly, the research was confined to private hospitals in Kampala, Uganda, which may not reflect the AMS practices and challenges in public healthcare facilities or in other regions of Uganda. Additionally, the study relied on self-reported data from healthcare staff and key informants, which may introduce recall bias or social desirability bias, potentially affecting the accuracy of responses. The cross-sectional nature of the study limits its ability to establish causality between AMS practices and specific outcomes, such as antimicrobial consumption or resistance patterns. Another limitation is the exclusion of patient perspectives, which could have provided a more holistic understanding of how patient expectations and satisfaction influence prescribing practices. Furthermore, the absence of a point prevalence survey (PPS) or longitudinal data on antimicrobial use restricts a comprehensive evaluation of AMS effectiveness over time. Finally, resource constraints limited the scope of diagnostic data analysis, particularly on bacterial resistance patterns. Future studies should adopt multi-centre designs, integrate public and private sectors, and employ mixed method approaches to capture a broader and deeper understanding of AMS implementation and its impact on antimicrobial resistance.

## Conclusions and recommendations

This study provides valuable insights into the status of antimicrobial stewardship programmes in private hospitals in Kampala, Uganda, shedding light on the challenges and opportunities within this critical area of healthcare. The results demonstrate that inadequate prioritisation, poor resource allocation, and fragmented AMS-IPC collaboration remain significant barriers to effective stewardship. The high costs of antibiotics, coupled with patient expectations, further complicate efforts to promote rational antimicrobial use. Despite these challenges, the study highlights areas for improvement, such as integrating AMS into governance structures, expanding diagnostic capacity, and enhancing monitoring and evaluation frameworks. The study also underscores the importance of aligning hospital-level AMS activities with national AMR action plans to create synergistic effects. Strengthening collaboration between hospitals, regulators, and policymakers will be essential to overcoming the barriers identified. Hospitals must adopt innovative strategies, including pooled procurement and digital tools for real-time data management, to enhance the sustainability of AMS programmes. Through coordinated, multi-sectoral efforts, private hospitals in Kampala can contribute meaningfully to the global fight against antimicrobial resistance. By addressing the gaps identified in this study and implementing targeted interventions, private healthcare institutions in Uganda have the potential to lead in AMR mitigation efforts. Future studies should explore the long-term impact of AMS activities in private hospitals and identify scalable models that can be replicated in other low- and middle-income settings. With sustained commitment from stakeholders at all levels, AMS can evolve from an aspirational goal into a routine aspect of patient care, safeguarding the efficacy of antibiotics for future generations.

## Declarations

This manuscript is partly based on Master of Science Degree thesis by Dr. Doris Kananu Kubai, the first author of this manuscript, submitted to Makerere University [66].

## Supporting information

S1 Checklist Inclusivity in global research. (DOCX)
